# Serum irisin levels and osteoporosis in patients with advanced chronic kidney disease and renal transplant recipients

**DOI:** 10.1007/s11255-023-03475-7

**Published:** 2023-02-11

**Authors:** Canan Demir, Ali Doğan Dursun, Gülçin Türkmen Sarıyıldız, Aykut İlker Arslan

**Affiliations:** 1 Department of Endocrinology and Metabolism, Atılım Üniversitesi Tıp Fakültesi, Kızılcaşarİncek Gölbaşı, 06830 Ankara, Turkey; 2 Department of Physiology, Medical School, Atilim University, and Vocational School of Health Services, Atilim University, Ankara, Turkey; 3 Department of General Surgery, Medicana International Ankara Hospital, and Operating Room Services, Vocational School of Health Services, Atılım University, Ankara, Turkey; 4 Department of Medical Laboratory, Vocational School of Health Services, Atılım University, Medicana International Ankara Hospital, Ankara, Turkey

**Keywords:** Bone mineral density, Irisin, Chronic kidney disease, Osteoporosis, Renal transplantation

## Abstract

**Aim:**

To elucidate the association of serum irisin levels with bone mineral density (BMD) and calcium–phosphorus metabolism parameters in chronic kidney disease (CKD) patients and renal transplant recipients (RTRs).

**Methods:**

This is a cross-sectional study involving CKD patients and RTRs. Healthy volunteers served as controls. Age, gender, and dialysis vintage were recorded. Serum irisin, creatinine, glucose, calcium, albumin, 25(OH) vitamin D, ferritin, C-reactive protein, A1C, and lipid profile were studied in all participants. Estimated glomerular filtration rate (eGFR), corrected calcium, and body mass index (BMI) were calculated.

**Results:**

Overall, 49 patients (23 hemodialysis, 26 RTRs) and 25 control subjects were included. In hemodialysis (HD) group, 8 patients (34.8%) had osteoporosis, and 12 patients (52.2%) had osteopenia. In RTR group, 3 patients (11.5%) had osteoporosis, while 15 patients (57.7%) had osteopenia. Among controls, one had osteoporosis, and 7 had osteopenia. There was no significant difference between HD and RTRs; however, osteoporosis rate was significantly lower in control subjects. BMD measurements (femur and lumbar T- and Z-scores) were comparable between HD and RTR groups. Control group DEXA values were similar to RTRs; however, they were significantly higher compared to HD group. 25(OH) vitamin D levels were comparable between the HD and RTR groups, and these were significantly lower compared to values of the control group. Mean serum irisin level was 426.6 ± 191.2 pg/mL in hemodialysis group, 342.6 ± 174.8 in the RTR group, and 208.0 ± 186.1 in controls. Serum irisin levels were similar in RTR and HD groups, but their values were significantly higher compared to controls. When we compared serum irisin levels between patients with and without osteoporosis in the whole cohort and hemodialysis and RTR groups, there was no difference. Serum irisin was positively correlated with lumbar T-score both in hemodialysis and RTR groups.

**Conclusion:**

Our study is the first in the literature revealing the positive correlation of serum irisin level with femur T-score in RTRs. Serum irisin level was also positively correlated with femur T-scores in hemodialysis patients.

## Introduction

Irisin is a member of the family known as myokines which are released from muscle in response to muscle contraction and allow muscle–bone and muscle–fat tissue cross-talk [1]. Irisin is produced by cleavage of the myocyte membrane protein FNDC5 and has a molecular weight between 10 and 32 kDa reported in various studies [2]. Irisin has a wide range of physiologic activities, including converting white fat to brown fat, improvement in insulin resistance and glucose tolerance, increasing nitric oxide release, and vasodilation, among others [3]. Serum irisin levels vary depending on many factors, including, but not limited to, sarcopenia, presence of diabetes mellitus, hypertension and chronic kidney disease, body mass index, and primary hyperparathyroidism [4–7].

The primary target of irisin is considered to be bone tissue. Osteocytes, osteoblasts and osteoclasts are all affected by irisin in some way. While osteocytes and osteoblasts are directly activated by irisin, osteoclasts interact in a dual way: direct activation by irisin and an indirect inhibition by irisin-induced osteoprotegerin [8, 9]. In the sum of these effects, irisin has been shown in experimental models to promote bone formation, prevent steroid-induced apoptosis, prevent disuse-induced loss of bone and muscle mass, and accelerates fracture healing [5]. Human data also confirmed the beneficial roles of irisin on bones. Several studies have demonstrated that serum irisin levels are positively correlated with bone mineral density in soccer players [10], older people [11], and postmenopausal women [12].

Chronic kidney disease (CKD) has detrimental effects on bones in the forms of osteoporosis, osteomalacia, and renal osteodystrophy [13]. Several studies to date have revealed decreased serum irisin levels in patients with chronic kidney disease [14]. Several factors were put forward to explain the positive correlation between glomerular filtration rate and serum irisin levels, including hyperparathyroidism, insulin resistance, sarcopenia, dialysis adequacy, and uremic toxins [15–17]. The association of serum irisin and bone mineral density has been studied poorly in patients with CKD [18]. Renal transplant recipients (RTRs) have a considerable burden of bone disease both related to renal osteodystrophy of the pretransplant dialysis phase and steroid-related osteoporosis of the transplantation phase [19]. As far as we know, no study to date has evaluated irisin and bone mineral density association in RTRs.

Hence, we aimed to elucidate the association of serum irisin levels with bone mineral density and other factors in a mixed cohort of hemodialysis patients and RTRs.

## Materials and methods

### Patients, design, and setting

This is a cross-sectional study in which we evaluated serum irisin levels along with calcium and phosphorus metabolism parameters and bone mineral density in chronic kidney disease patients and renal transplant recipients (RTRs). We also included age- and sex-matched healthy volunteers as the control group. The study was conducted at the nephrology and transplantation unit of Medicana Hospital, Ankara, Turkey. Patient recruitment lasted from 1 January 2022 to 30 April 2022. Patients were selected among consecutive patients who were evaluated at an outpatient nephrology clinic and willing to participate in the study. Renal transplant recipients who were being followed at the transplantation unit and willing to take part in the study were also included. Subjects in the control group were selected among patients who presented to the check-up outpatient clinic who were not known to have kidney disease or osteoporosis. Patients with one or more of the following characteristics were excluded from the study: receiving osteoporosis treatment with medications such as bisphosphonates, teriparatide, or estrogen, history of lumbar vertebral fracture and/or instrumentation, history of malignancy with bone metastasis, and refusal to participate in the study. The Medicana Hospital's ethics committee approved the study protocol (BŞH.2022/12). Study aims and protocol were explained in detail to all prospective participants of the study, and written informed consent forms were signed by those who consented to participate. This study procedure was carried out in accordance with the Declaration of Helsinki.

### Data collection

Age, gender, body height and weight, duration of chronic kidney disease, dialysis vintage, and duration of renal transplantation status were recorded for study participants. Drug uses related to calcium-phosphorus metabolism such as active vitamin D, cholecalciferol and/or phosphate binders were determined for each patient.

### Irisin and other biochemical measurements

Fasting blood samples were taken from all study participants (30 min before starting hemodialysis in maintenance dialysis patients) and centrifuged at 3000 g for 10 min. Serum samples were used to make biochemical measurements, including creatinine, glucose, calcium, albumin, 25(OH) vitamin D, ferritin, C-reactive protein, hemoglobin A1C, low-density lipoprotein (LDL), and high-density lipoprotein (HDL) cholesterol, triglycerides by a clinical chemistry analyzer (Abbott ARCHITECT c8000). Complete blood counts were also studied in all participants. Serum intact parathyroid hormone level was measured by enzyme-linked immunosorbent assay (ELISA) (Abbott ARCHITECT i2000SR immunoassay analyzer). A commercial ELISA assay was used to measure serum irisin levels (Elabscience, Texas, US). Detection range is reported by the manufacturer as between 15.63 and 1000 pg/mL. The manufacturer claims that no significant cross-reactivity or interference between Human Irisin and analogs was observed. We found intra- and inter-assay coefficient variation of irisin to be < 10%.

The estimated glomerular filtration rate (eGFR) was calculated by the Modification of Diet in Renal Disease (MDRD) equation [20]. Corrected calcium was calculated by the following formula:

Corrected calcium (mg/dL) = measured total Ca (mg/dL) + 0.8 (4.0 − serum albumin [g/dL]).

Kt/V, URR (urea reduction ratio) and nPCR (Normalized Protein Catabolic Rate) were calculated via online calculators for each hemodialysis patient.

Body mass index (BMI) was calculated by dividing weight in kilograms by height in meters squared. Homeostatic Model Assessment for Insulin Resistance (HOMA-IR) was calculated by the following equation: Score = (Fasting insulin) × (Fasting glucose)/405 [21].

### Bone mineral density measurements

All patients and control subjects underwent lumbar and femur neck region bone mineral density measurements by dual-energy X-ray absorptiometry (DEXA) (Hologic QDR Explorer, Bedford, MA, USA). T- and Z-scores were calculated for all measurements. A T-score of -2.5 or below was accepted as osteoporosis. T-scores between -1 and -2.5 was considered osteopenia [22].

### Statistical analysis

The Shapiro–Wilks test, histogram, and Q–Q plot were used to evaluate the normality. Normally distributed continuous variables were expressed as mean ± standard deviation, and non-normally distributed variables were specified as median (min–max). Categorical variables were given as numbers and percentages. Chi-square test or Fisher's exact test was used to compare categorical variables between the groups. In numerical variables, to compare two-group comparisons, the independent *t*-test or the Mann–Whitney *U* test were used, whereas, for three groups comparison, the one-way analysis of variance (ANOVA) or Kruskal–Wallis test was used. The Spearman correlation and point pairwise correlation test were performed to seek the correlation.

We performed univariate and multivariate linear regression analyses to determine the independent associates of the lumbar T-score. Based on the results of univariate linear regression, among those with a *P* value less than 0.05 and the variables with clinical significance were included in the multivariate linear regression analysis.

The SPSS 25.0 software package (IBM, Armonk, NY, USA) was used for analysis. A probability value of *P* < 0.05 was considered statistically significant, and two-tailed *P* values were used for all statistics.

## Results

A total of 49 patients were included in this cross-sectional study. Of these, 23 patients were undergoing maintenance hemodialysis, while 26 patients were renal transplant recipients. Twenty-five healthy participants served as the control group. The median dialysis vintage was 4 years. The median duration since transplantation was 3 years among RTRs. All three groups were matched in terms of age and sex distribution. All hemodialysis patients except one had arteriovenous fistula, one patient had tunneled double lumen dialysis catheter. Table 1 summarizes clinicodemographic features, laboratory parameters and DEXA measurements of the hemodialysis, renal transplant recipient and the control groups.

**Table 1:** Demographic characteristics, routine biochemistry, insulin resistance, calcium–phosphorus metabolism parameters, and bone mineral density measurements in hemodialysis patients and RTRs and control subjects

	Hemodialysis (*n* = 23)	Renal transplant recipients (*n* = 26)	Controls (*n* = 25)	*p* value
Age (years)	42.0 ± 14.0	45.8 ± 10.1	39.4 ± 8.0	0.051
Sex				
Female	9 (39.1%)	6 (23.1%)	14 (56.0%)	0.055
Male	14 (60.9%)	20 (76.9%)	11 (44.0%)	
Body mass index (kg/m^2^)	25.3 ± 3.9	26.1 ± 2.8	25.6 ± 4.4	0.731
C-reactive protein (mg/dL)	3.1 (0.1–23.1)	0.2 (0.1–62.5)	0.0 (0.0–0.7)	** < 0.001**
eGFR (mL/min)	7.2 ± 2.1	58.6 ± 14.8	98.9 ± 16.6	** < 0.001**
Creatinine (mg/dL)	5.9 ± 3.5	1.4 ± 0.4	0.8 ± 0.2	** < 0.001**
HDL cholesterol (mg/dL)	44.0 ± 13.9	55.9 ± 12.7	47.2 ± 10.1	**0.007**
Triglycerides (mg/dL)	162.0 ± 111.4	151.2 ± 63.4	123.9 ± 109.6	0.453
LDL cholesterol (mg/dL)	95.2 ± 33.5	125.2 ± 36.9	128.2 ± 32.6	**0.002**
Total protein (g/dL)				
Albumin (g/dL)	4.4 ± 0.4	4.6 ± 0.4	4.6 ± 1.0	**0.048**
Dialysis related parameters				
Kt/V	1.66 ± 0.19	–	–	**–**
URR (%)	75 ± 5.2	–	–	**–**
nPCR	1.278 ± 0.282	–	–	**–**
Bone turnover and calcium–phosphorus parameters				
Alkaline phosphatase (IU/L)	86.0 (40.0–309.0)	63.5 (42.0–187.0)	56.0 (30.0–76.0)	**0.001**
Corrected calcium (mg/dL)	8.4 (7.1–9.8)	8.9 (5.1–11.6)	9.3 (4.4–10.0)	**0.001**
Phosphorus (mg/dL)	4.7 ± 1.7	3.4 ± 0.7	3.4 ± 0.4	**0.005**
25(OH) Vitamin D (ng/mL)	12.0 (5.0–103.0)	12.0 (5.0–42.0)	21.2 (8.0–47.0)	**0.003**
Parathyroid hormone (pg/mL)	327.4 (23.7–1755.7)	66.4 (9.7–489.5)	44.9 (19.4–82.4)	** < 0.001**
Bone mineral density (DEXA)				
Femoral neck T-score	− 1.5 (− 3.3 to 4.0)	− 0.9 (− 2.8 to 1.2)	− 0.2 (− 2.0 to 1.1)	**0.001**
Femoral neck Z-score	− 1.2 (− 2.9 to 5.1)	− 0.8 (− 2.1 to 2.2)	− 0.1 (− 1.4 to 1.4)	**0.006**
Femoral total T-score	− 1.6 (− 2.9 to 1.5)	− 1.4 (− 2.8 to 1.0)	− 0.7 (− 2.5 to 0.9)	**0.006**
Femoral total Z-score	− 1.4 (− 2.9 to 2.3)	− 1.2 (− 2.6 to 1.3)	− 0.5 (− 2.1 to 1.0)	**0.007**
Lumbar T-score	− 1.8 (− 4.2 to 2.7)	− 1.1 (− 4.6 to 2.1)	− 0.7 (− 2.2 to 1.4)	**0.006**
Lumbar Z-score	− 1.7 (− 4.1 to 3.8)	− 1.0 (− 4.5 to 1.9)	− 0.6 (− 2.0 to 1.7)	**0.007**
Glucose tolerance and insulin resistance				
Fasting glucose (mg/dL)	87.0 (76.0–202.0)	91.0 (30.0–231.0)	77.0 (64.0–295.0)	** < 0.001**
HbA1c (%)	4.8 (4.1–7.5)	5.6 (4.9–9.3)	5.3 (4.4–8.8)	** < 0.001**
Insulin (μIU/mL)	7.4 (3.6–35.6)	9.9 (2.2–36.2)	9.3 (3.6–18.1)	0.414
HOMA-IR	1.6 (0.7–10.6)	2.2 (0.6–16.7)	1.7 (0.7–12.1)	0.376
Bone mineral status				
Normal	3 (13.0%)	8 (30.8%)	17 (68.0%)	
Osteopenia	12 (52.2%)	15 (57.7%)	7 (28.0%)	**0.001**
Osteoporosis	8 (34.8%)	3 (11.5%)	1 (4.0%)	
Serum irisin (pg/mL)	426.6 ± 191.2	342.6 ± 174.8	208.0 ± 186.1	**0.001**

*p* value < 0.05 is statistically significant

eGFR: estimated glomerular filtration rate, HDL cholesterol: High-density lipoprotein cholesterol, LDL cholesterol: Low-density lipoprotein cholesterol, URR: Urea reduction ratio, nPCR: normalized protein catabolic rate, Post-hoc comparison with Bonferroni correction: C-reactive protein: control group vs hemodialysis patients, control group vs transplant recipients, eGFR: control group vs hemodialysis patients, control group vs transplant recipients, hemodialysis patients vs transplant recipients, Creatinine: control group vs hemodialysis patients, hemodialysis patients vs transplant recipients, HDL cholesterol: control group vs transplant recipients, hemodialysis patients vs transplant recipients, LDL cholesterol: control group vs hemodialysis patients, hemodialysis patients vs transplant recipients, Albumin: There were no differences after Bonferroni correction, Alkaline Phosphatase: control group vs hemodialysis patients, Corrected Calcium: control group vs hemodialysis patients, Phosphorus: control group vs hemodialysis patients, hemodialysis patients vs transplant recipients, 25(OH) Vitamin D: control group vs hemodialysis patients, control group vs transplant recipients, Parathyroid hormone: control group vs hemodialysis patients, control group vs transplant recipients, hemodialysis patients vs transplant recipients, Femoral neck T-score: control group vs hemodialysis patients, Femoral neck Z-score: control group vs hemodialysis patients, Femoral total T-score: control group vs hemodialysis patients, Femoral total Z-score: control group vs hemodialysis patients, Lumbar T-score: control group vs hemodialysis patients, Lumbar T-score: control group vs hemodialysis patients, Fasting Glucose: control group vs hemodialysis patients, control group vs transplant recipients, HbA1c: control group vs hemodialysis patients, hemodialysis patients vs transplant recipients, Irisin: control group vs hemodialysis patients, control group vs transplant recipients, Bone Mineral Status: The incidence of osteoporosis was higher in hemodialysis than in controls, there was no difference in terms of osteopenia in the three groups

### Parameters of calcium and phosphorus metabolism and bone mineral density

In the hemodialysis group, 8 patients (34.8%) had osteoporosis, and 12 patients (52.2%) had osteopenia. In the RTR group, only 3 patients (11.5%) had osteoporosis, while 15 patients (57.7%) had osteopenia. Among control subjects, one participant (4%) had osteoporosis; whereas, 7 people (28%) had osteopenia. Although numerically, osteoporosis was more common among hemodialysis patients, there was no significant difference between the groups in this regard (*P* = 0.086). In the control group, only one subject had osteoporosis. Bone mineral density measurements were comparable between the hemodialysis patients and RTRs. All T and Z-scores in femoral and lumbar regions were significantly higher in control subjects compared to hemodialysis patients. There was no significant difference between RTRs and control subjects in this regard (Table 1).

Serum corrected calcium levels were significantly lower in hemodialysis patients compared to RTRs and control subjects, and the median values were similar in the latter two groups. Serum parathormone levels were highest in hemodialysis patients and lowest in control subjects. Median PTH values were significantly different in all three groups. Serum C-reactive protein, alkaline phosphatase, corrected calcium, fasting glucose, albumin, 25(OH) vitamin D, and irisin levels were comparable between the hemodialysis and RTR groups.

### Serum irisin levels

**Fig. 1:**
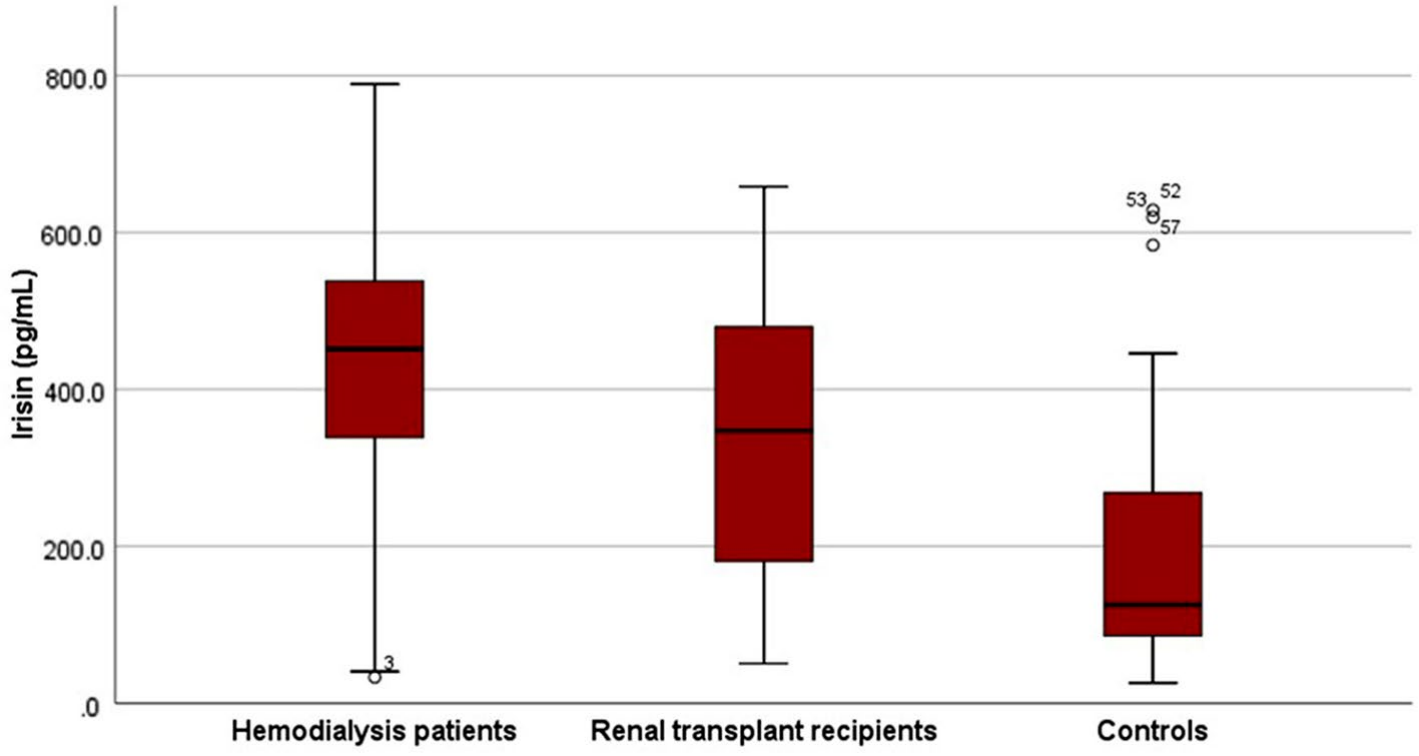
Box plot displays the median serum irisin levels of the groups

The median serum irisin level was 426.6 ± 191.2 pg/mL in the hemodialysis group and 342.6 ± 174.8 ng/mL in the RTR group. There was no difference between the groups in this respect (Table 1). Control subjects had significantly lower serum irisin levels compared to hemodialysis patients and RTRs (Fig. 1). When we compared serum irisin levels between patients with and without osteoporosis in the whole cohort and hemodialysis and RTR groups, there was no difference in any of the comparisons. In all three comparisons, serum irisin levels were comparable between patients with and without osteoporosis (Table 2).

**Table 2:** Serum irisin level comparison between patients with and without osteoporosis in the whole group, RTR group, and hemodialysis group

	Osteoporosis absent	Osteoporosis present	*p* value
All patients	(*n* = 38)	(*n* = 11)	
Serum irisin level (pg/mL)	405.4 ± 170.7	301.2 ± 219.9	0.102
Hemodialysis patients	(*n* = 15)	(*n* = 8)	
Serum irisin level (pg/mL)	480.0 ± 149.3	326.2 ± 229.7	0.118
Transplantation patients	(*n* = 23)	(*n* = 3)	
Serum irisin level (pg/mL)	356.8 ± 169.1	233.8 ± 2183	0.260

Among hemodialysis patients, serum irisin level showed a moderately strong positive correlation with femur neck T-score (*r* = 0.421, *P* = 0.046) and femur neck Z-score (*r* = 0.476, *P* = 0.022). İrisin was also significantly and positively correlated with BMI (*r* = 0.450, *P* = 0.031). In the RTR group, serum irisin level was significantly correlated with femur neck T-score (*r* = 0.410, *P* = 0.037). In the healthy controls group irisin level was not correlated with lumbar T-score (*r* = 0.224, *P* = 0.283). Similarly, lumbar T-score and serum irisin level had correlation neither in hemodialysis nor in RTRs groups (*r* = 0.375, *P* = 0.078 and *r* = 0.140, *P* = 0.497, respectively).

### Independent associates of femur T-score

In the RTR group, irisin appeared as a significant associate with femur T-score. However, in the multivariate logistic regression model, only body mass index was an independent and significant associate of femoral T-score (Table 3) Multivariate analysis was not performed in hemodialysis patients since serum irisin did not appear as a significant determiner of femur neck or total T-score.

**Table 3:** Univariate and multivariate linear regression models to determine the independent associates of the femoral neck T-score in the RTR group

	Univariate LR			Multivariate LR		
Variables	OR	95% CI	*p* value	OR	95% CI	*p* value
Sex	0.653	− 0.549 to 1.855	0.273	–	–	–
Body mass index	0.202	0.035–0.368	**0.020**	0.177	0.018–0.337	**0.031**
Irisin	0.003	0.001–0.002	**0.032**	0.002	0.001–0.005	0.057
Parathormone	0.003	− 0.003 to 0.008	0.349	–	–	–
Time since transplantation	− 0.002	− 0.148 to 0.144	0.982	–	–	–
25(OH) vitamin D	− 0.010	− 0.076 to 0.056	0.755	–	–	–
HOMA_IR	− 0.038	− 0.108 to 0.103	0.580	–	–	–

*p* value < 0.05 is statistically significant

*OR* Odds Ratio, *CI* Confidence Interval, *LR* Linear Regression

## Discussion

The major findings of the present study were as follows: (1) Serum irisin levels were significantly lower in control subjects compared to hemodialysis patients and RTRs. (2) Although eGFR was significantly lower in hemodialysis patients compared to RTRs, serum irisin levels were comparable between the two groups. (3) Serum irisin level was not different between patients with and without osteoporosis in RTRs and hemodialysis patients. (3) Serum irisin level, similar to previous reports, was positively correlated with femur T-score in hemodialysis patients. (4) For the first time in the literature, our results showed a significant positive correlation between serum irisin level and femur T-score in renal transplant recipients. (5) Serum irisin level was an independent associate of femur T-score neither in hemodialysis nor in the RTR group.

Osteoporosis is a metabolic bone disorder characterized by reduced bone strength and architecture leading to increased fracture risk [23]. Osteoporosis seems like one of the inevitable consequences of advanced kidney disease. Several diverse factors contribute to the development of osteoporosis in patients with CKD, including hypocalcemia, hyperphosphatemia, active vitamin D deficiency, increased parathormone, increased fibroblast growth factor 23, among others [24]. Unfortunately, osteoporosis does not remain silent in CKD patients and, due to fractures, is associated with increased morbidity and mortality [25]. It was reported that between 9.5 and 23% of hemodialysis patients had osteoporosis [26].

The presence of renal osteodystrophy (ROD) complicates the diagnosis of osteoporosis in patients with advanced CKD: bone turnover markers, particularly parathormone, show considerable inconsistencies relative to the underlying bone pathology of the renal osteodystrophy. DEXA also has some shortcomings in patients with advanced CKD. It cannot determine a specific pathologic subtype of ROD and might be affected by increased skeletal and extraskeletal calcification commonly seen in CKD patients [27]. Thus, it is still an ongoing need to find a reliable marker to inform us regarding the presence of osteoporosis in patients with CKD. İrisin, in this respect, might be a candidate marker to detect and evaluate bone mineral abnormalities in CKD patients.

Several studies in the healthy population have shown that irisin is positively correlated with bone mineral density markers and T-scores [10, 11, 28, 29]. A meta-analysis including 1,018 middle-aged and older adults showed that the presence of osteoporosis was associated with lower serum irisin levels. Moreover, postmenopausal women and those with a fracture history had even lower levels of serum irisin. In patients with rheumatoid arthritis, reduced irisin levels could predict vertebral fractures independently [30]. Similar results were replicated in postmenopausal women as well [31]. Considering the major morbidity and mortality, it burdens on the CKD population; the osteoporosis–irisin association has not been adequately studied. Lu and colleagues evaluated serum irisin levels and bone mineral density in 80 maintenance hemodialysis patients [18]. The authors assessed bone density with DEXA at L2–L4 vertebrae. Of all patients, 12.5% had osteoporosis based on lumbar T-scores. T-score was found to be positively correlated with serum irisin level. Our results also confirmed the results of the latter study. Lumbar T-score was positively correlated with serum irisin level.

Several studies conducted in patients with CKD showed that eGFR is associated with serum irisin levels such that as eGFR decreases, serum irisin level decreases as well. Rodríguez-Carmona et al. evaluated 95 dialysis (hemodialysis and peritoneal dialysis) and predialysis patients. The authors found reduced serum irisin levels in CKD patients. However, levels of irisin were higher compared to those receiving hemodialysis or conservatively managed predialysis patients [32]. In a cross-sectional case–control study, Wen and colleagues found that serum irisin levels were lower in stage-5 CKD patients compared to normal subjects. Serum irisin levels showed an inverse correlation with serum creatinine values [33]. Our results did not demonstrate a significant difference in serum irisin levels between hemodialysis patients and RTRs. This result seems surprising, given the mean eGFR values were significantly different between the groups. We predicted that serum irisin levels would be lower in hemodialysis patients who had much lower eGFR values compared to healthy controls and RTRs. Since serum irisin levels are affected by numerous factors, the lack of difference in our results might be attributed to not controlling all relevant factors. However, serum irisin levels in healthy control subjects was significantly lower compared to values observed in hemodialysis patients and RTRs. Our results seem counterintuitive at first sight with regards to the results of the irisin studies published till now. One of the possible explanations might be the relatively small number of subjects in each subgroup of the patients in the present study. Another possible confounder is that serum irisin levels are affected by many variables. Some of these factors are diabetes mellitus, nonalcoholic fatty liver disease, cardiovascular disease and obesity, among others [34]. We could not control all these factors in our study. Another possibility explaining the divergent results is the inherent problems with ELISA assay of irisin. Albrecht and colleagues in their elaborate study examined different antibodies used in commercial irisin ELISA kits and questioned the reliability and validity of serum measurements performed with these kits. Thus, differing irisin kits might be responsible for contradicting results in the literature.

Some clinical studies revealed that serum irisin levels are also significantly and independently related to insulin resistance in patients with advanced kidney disease [15, 35]. In contrast to these studies, our results did not show any association of serum irisin levels with serum glucose or HOMA-IR values.

Renal transplant patients have higher rates of osteoporosis. In a recent study by Yavuz et al., it was shown that the prevalence of osteoporosis was 20% among RTRs. In contrast to the reversal of metabolic abnormalities related to uremia, bone changes usually remain stable and do not improve after renal transplantation [36]. Thus, osteoporosis related fractures are a common cause of morbidity among RTRs [37]. As far as we know, no study in the literature evaluated serum irisin levels in RTRs in the context of osteoporosis. Our results showed a significant and positive correlation between femur T-score and serum irisin level. However, serum irisin levels were not different from those of hemodialysis patients.

Some limitations of the present study deserve mention. First, our sample size was relatively small to reveal subtle differences between the hemodialysis and renal transplant group. Second, we could not determine and control all factors that can potentially affect serum irisin levels, such as underlying cardiovascular disease, sarcopenia, blood pressure, and daily activity level. Hence, these uncontrolled factors might have had an impact on our results.

In conclusion, despite the aforementioned limitations, our study is the first in the literature revealing the positive correlation of serum irisin level with femur T-score in renal transplant recipients. We also confirmed the results of a previous study in that serum irisin level was also positively correlated with femur T-scores in hemodialysis patients. Serum irisin level was found to be increased both in hemodialysis patients and RTRs relative to healthy control subjects. Since a number of factors can potentially affect serum irisin levels, to elucidate its precise role in osteoporosis in patients with CKD and renal transplantation, studies with large sample sizes and taking confounding factors into account are necessary.

## Data Availability

The data that support the findings of current study are available from the corresponding author, upon reasonable request.
